# Clinical Characteristics and Outcomes of Patients with Well-Differentiated Papillary Peritoneal Mesothelial Tumors

**DOI:** 10.1245/s10434-024-16004-2

**Published:** 2024-08-21

**Authors:** Michael Offin, Nicole Aguirre, Soo Ryum Yang, Jennifer L. Sauter, Georgios Karagkounis, Mostafa Mohamed, Andrea Cercek, Ritika Kundra, Yanming Zhang, Hui Mei Wang, Marty P. Morris, Marc Ladanyi, Garrett M. Nash, Marjorie G. Zauderer

**Affiliations:** 1 Thoracic Oncology Service, Department of Medicine, Memorial Sloan Kettering Cancer Center, New York, NY USA; 2 Weill Cornell Medical College, New York, NY USA; 3 Department of Colorectal Surgery, Memorial Sloan Kettering Cancer Center, New York, NY USA; 4 Department of Pathology, Memorial Sloan Kettering Cancer Center, New York, NY USA; 5 Gastrointestinal Oncology Service, Department of Medicine, Memorial Sloan Kettering Cancer Center, New York, NY USA

**Keywords:** Well-differentiated papillary peritoneal mesothelial tumors, Mesothelioma, Next-generation sequencing, Outcomes, Treatment

## Abstract

**Purpose:**

Well-differentiated papillary peritoneal mesothelial tumors (WDPMTs) are understudied and discrete from peritoneal mesotheliomas (PMs). We report clinicopathologic characteristics and outcomes of a large prospective WDPMT cohort.

**Methods:**

Patients with WDPMT identified between August 2007 and December 2020 were followed through January 2023. Clinical characteristics and outcomes were annotated. Overall survival (OS) was assessed from pathologic diagnosis. Germline variants were analyzed, and targeted next-generation sequencing (NGS; MSK-IMPACT) data were compared to PMs and diffuse pleural mesotheliomas (DPMs).

**Results:**

Among 54 patients, median age at diagnosis was 55 (range 20–76), 50% were female (*n* = 27), and 46% were smokers (*n* = 25; median 8 pack/years). Most (94%, *n* = 51) WDPMTs were found during surgical explorations for other indications, primarily other malignancies. Two patients underwent surgical resection for WDPMT; none received systemic therapy for WDPMT. Median OS was not reached (19/54; median follow up 4.5 years). Somatic NGS was available for 35% (19/54) of patients. *TRAF7* alterations were enriched in WDPMT (89%; 17/19) compared with PM (0%; 0/50; *p* < 0.0001) and DPM (0%; 0/74; *p* < 0.0001). In WDPMT compared with PM and DPM, there were less *BAP1* (0% [0/0] vs. 4% [8/50] vs. 46% [34/74]; *p* = 0.001 and *p* < 0.0001, respectively) and *NF2* (0% [0/0] vs. 24% [12/50] vs. 31% [23/74]; *p* = 0.03 and *p* = 0.001 respectively) alterations. Pathogenic germline variants were present in 23% (4/17) of WDPMTs.

**Conclusions:**

Well-differentiated papillary peritoneal mesothelial tumors were primarily incidental findings. There was no WDPMT-related mortality, so there was no distinct role for routine cytoreductive surgery or systemic therapy. Genomic profiles can help to differentiate WDPMT from DPM and PM.

**Supplementary Information:**

The online version contains supplementary material available at 10.1245/s10434-024-16004-2.

Well-differentiated papillary peritoneal mesothelial tumors (WDPMTs) represent a discrete abdominal proliferative process defined by archetypal histologic and genomic findings and a relatively indolent course.^1–4^ Until recently, WDPMT was considered to be on a spectrum with peritoneal mesotheliomas (PMs); however, this association is now unclear. In rare cases where a patient develops PM in the setting of a previous papillary peritoneal tumor, the initial neoplasm is more likely a papillary mesothelioma in situ (PMIS; a lesion with invasive potential) rather than WDPMT.^5^ While there are several approved and recommended treatments for diffuse pleural mesotheliomas (DPMs) and NCCN (National Comprehensive Cancer Network) recognized options for PM, management of WDPMT is undefined and inappropriately extrapolated from PM.

There is a paucity of data on the genomic landscape of WDPMT compared with the burgeoning knowledge around DPM and PM.^2,6,7^ Also, germline alteration rates are established in patients with PM and DPM but not in patients with WDPMT.^7,8^ Given the clinically distinct nature of WDPMT, a better understanding of the genomics of the disease could help to differentiate it from mesothelioma to ensure a correct diagnosis and guide patient care.^1–4^ We report the distinct clinicopathologic characteristics and outcomes of a large prospective cohort of patients with WDPMT.

## Methods

Patients evaluated at Memorial Sloan Kettering Cancer Center (MSK) between August 2007 and December 2020 with a pathologic diagnosis of WDPMT were annotated. Clinicopathologic features (age, sex, race, smoking status, previous cancers, and treatment history) and median overall survival (OS) from the date of initial diagnosis were assessed through review of the Electronic Medical Record through January 2023. Given the purported long survival of patients with WDPMT, an extended follow-up interval was necessary.^3,4^ All cases were reviewed by a multidisciplinary mesothelioma team: medical oncologists, gastrointestinal surgeons, pathologists, and molecular pathologists (when applicable). The study was approved by the MSK Institutional Review Board (IRB) and conducted in accordance with the United States Common Rule.

All patients with available material and matched baseline normal control blood underwent targeted somatic next-generation sequencing (NGS) on the MSK-IMPACT platform^9^. Somatic alterations were analyzed. In patients with other malignancies, samples were selected from the tissue block from the WDPMT specimen and reviewed to ensure no pathognomonic somatic alterations were noted indicative of other sites of origin. Cases with an aggressive disease course requiring disease-specific treatment or discordant molecular findings (such as the absence of *TRAF7* or presences of *BAP1*, *CDKN2*, *NF2*) were re-reviewed by the study pathologist and molecular oncologist.^10^ Germline variants were analyzed in patients who provided written informed consent.^8^

If genomic results were ambiguous (e.g., possessed genomic alterations associated with PM), additional confirmational testing was used. Fluorescence in situ hybridization (FISH) analysis using CDKN2A (9p21) and centromere specific chromosome 9 (CEP9) probes (both from Abbott Molecular, Des Plains, IL) and immunohistochemistry (IHC) on formalin-fixed paraffin-embedded tissue using MTAP monoclonal antibody clone 1813 (NBP2-75731; Novus Biologicals, Centennial, CO) were used to characterize functional CDKN2 expression.

Somatic alterations in WDPMT samples were compared with PM in our published cohort and DPM in a publicly available data set (The Cancer Genome Atlas [TCGA]) using the Fisher’s exact test.^6,7^

## Results

We identified 54 patients with WDPMT for evaluation (Table 1). The median age at diagnosis was 55 (range 20–76) years, 50% were female, and 46% were former smokers (median of 8 pack years). Asbestos exposure was not uniformly collected and cannot be commented upon. Most diagnoses resulted from surgical interventions performed for other malignancies (72%; 39/54) or other benign medical conditions (22%; 12/54). The most common co-occurring cancers were gastrointestinal (GI) and prostatic in origin. Twelve patients required systemic cytotoxic chemotherapy for the other malignancy. All (6%; 3/54) patients who underwent initial diagnostic procedures for WDPMT did so in the setting of a solitary abdominal nodule of unclear significance.

**Table 1:** Patient characteristics in the 54 patients with well-differentiated papillary peritoneal mesothelial tumors (WDPMTs)

Patient characteristics	WDPMT (*n* = 54)
Median age at diagnosis (years, range)	55 (20–76)
*Sex*	
Male, *n* (%)	27 (50)
Female, *n* (%)	27 (50)
*Self-identified race*	
Caucasian, *n* (%)	41 (76)
African American, n (%)	5 (9)
Asian, *n* (%)	5 (9)
Other/not disclosed, *n* (%)	3 (6)
*Smoking status*	
Never, *n*	29
Ever, n (pack-years; range)	25 (8; 3–50)
*Incidentally diagnosed during other surgical procedure*	
No, *n* (%)	3 (6)
Yes, *n* (%)	51 (94)
*Initial diagnostic surgery performed for:*	
GI cancers, *n* (%)	16 (30)
Colorectal cancer, *n* (%)	10 (19)
Esophageal cancer, *n* (%)	1 (2)
Gastric cancer, *n* (%)	2 (4)
Pancreatic cancer, *n* (%)	3 (6)
Metastatic breast cancers, n (%)	2 (4)
Ovarian neoplasms, n (%)	4 (7)
Prostate cancers, *n* (%)	13 (24)
Renal cell carcinomas, *n* (%)	2 (4)
Other malignancies, *n* (%)	2^a^ (4)
Non cancer related surgeries	12^b^ (22)
*Disease-specific treatment*	
Surgical debulking for WDPMT, *n* (%)	2 (4)
Systemic therapies for WDPMT, *n* (%)	0

*WDPMT* well-differentiated papillary peritoneal mesothelioma tumors

^a^One liposarcoma and one gastrointestinal stromal tumor (GIST)

^b^One appendectomy, four abdominal hysterectomies, one myomectomy, two cholecystectomy, two sleeve gastrectomy, two diagnostic laparoscopies

No patients required systemic therapy for WDPMT. Two patients underwent cytoreductive surgery for WDPMT: one for patient preference in the setting of multiple abdominal implants, and one for initial concern for subcentimeter growth on interval cross-sectional imaging. One patient received a hemicolectomy owing to a concerning nodule on imaging, which was WDPMT. These patients were alive >10 years after surgical intervention. No patients with pathologically confirmed WDPMT had intercurrent PM during follow-up. With a median follow-up of 4.5 (range 0.1–14.2) years, including one patient lost to follow-up soon after initial visit, the median OS was not reached. There was no WDPMT-specific mortality (Supplemental Fig. 1A). Median OS did not differ and was not reached for the 16 patients with no co-occurring cancer diagnoses (13 diagnosed during noncancer-related surgeries and 3 with WDPMT only) and for the 38 patients with other malignancies (hazard ratio 3.89, 95% confidence interval [CI] 0.80–18.88. *p* = 0.09; Supplemental Fig. 1B).

**Fig. 1:**
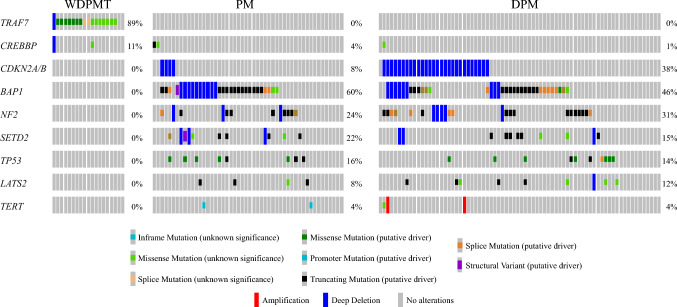
Oncoprint comparing the genomic landscapes of patients with well-differentiated papillary peritoneal mesothelial tumors (WDPMT; *n* = 19) versus previously published data from patients with peritoneal mesotheliomas^7^ (PM; *n* = 50) and diffuse pleural mesotheliomas^6^ (DPM; The Cancer Genome Atlas [TCGA]; *n* = 74)

**Fig. 2:**
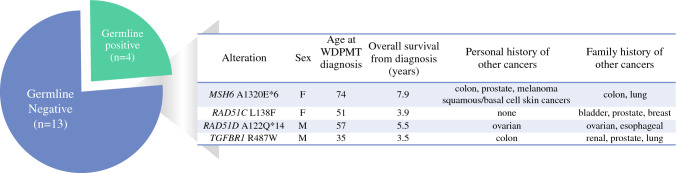
Characteristics and first-degree family history of cancers in patients with well-differentiated papillary peritoneal mesothelial tumors (WDPMT) and germline mutations detected by next-generation sequencing of biopsy samples

Next-generation sequencing was performed on all usable material (35% (19/54) of WDPMT) (Fig. 1; Supplemental Table 1). Several samples were excluded because of insufficient material or lack of matched normal blood. Overall, 89% (17/19) had a *TRAF7* alteration within the WD40-domain (Supplemental Fig. 2) and 11% (2/19) had a *CREBBP* alteration. Samples without a *TRAF7* alteration were manually reviewed by a molecular pathologist and were found to have adequate cellularity and no relevant intronic/nonexonic mutations. One sample from a patient with pathologically confirmed WDPMT had a potential *CDKN2A* deletion; however, confirmational FISH and MTAP IHC indicated no functional CDKN2 loss. Of the 17 patients who consented for germline testing, 23% (n = 4) had inherited pathogenic alterations, including MSH6 A1320E*6, *RAD51C L138F*, RAD51D A122Q*14, and *TGFBR1 R478W* (Fig. 2).

The genomic landscape of WDPMT differed substantially compared with PM with similar demographic characteristics (Supplemental Table 2) and DPM (Supplemental Table 3).^6,7^ Relative to PM, WDPMT lacked *BAP1* (0/19 vs. 30/50, *p* = 0.001, *NF2* (0/19 vs. 12/50, *p* = 0.03), and *SETD2* (0/19 vs. 11/50, *p* = 0.03) alterations. Relative to DPM, WDPMT lacked alterations in *CDKN2A/B* (0/19 vs 28/74, *p* = 0.0005), *BAP1* (0/19 vs. 34/74, *p* < 0.0001), and *NF2* (0/19 vs. 23/74, *p* = 0.001). *TRAF7* alterations only occurred in WDPMT samples (17/19 vs. 0/50 [PM] vs. 0/74 [DPM], both *p’s < 0.001*).

## Discussion

Well-differentiated papillary peritoneal mesothelial tumors exhibit unique clinical, pathologic, and genomic features relative to mesotheliomas. *TRAF7* was enriched in WDPMT, while common mesothelioma alterations (*BAP1*, *NF2*, *CDKN2*, and *SETD2*) were absent. No patients required WDPMT-directed systemic therapy or interventions for symptom control, and there was no disease-specific mortality observed throughout the long duration of follow-up. No cases of WDPMT transformed to PM, further suggesting that WDPMT is biologically distinct from mesothelioma.^5^

Most WDPMTs were found during procedures for other malignancies (most commonly GI and prostate cancers). It is unclear whether WDPMT is associated or shares underlying pathology with other abdominal malignancies; larger multisite cohort reviews are needed to further evaluate this. While we noted a trend toward inferior OS in patients with concurrent malignancies, the survival of this cohort was remarkably robust, with no WDPMT-specific mortality during a median 4.5-year follow-up period. These outcomes mirror other studies,^3,11^ which included patients who received heterogeneous treatments for WDPMT, including surgery, chemotherapy, and/or radiotherapy, suggesting that anticancer therapy does not augment the already good outcomes of this condition.

Enrichment of *TRAF7* alterations primarily confined to the WD-40 domain was a defining feature of WDPMT.^2^ While *TRAF7* was only found in WDPMT, not all WDPMT had a *TRAF7* alteration; two cases had wildtype *TRAF7*. Previous studies also have reported enrichment of *TRAF7* and *CDC42* in WDPMT and, similar to our results, reduced prevalence of common alterations seen in mesotheliomas, such as *BAP1, NF2, CDKN2A/B, TP53,* and *SETD2.*^10^ *CDC42* is mutually exclusive of *TRAF7* but was not included in our NGS assay.^10^ This genomic landscape in combination with expert pathology review to confirm histologic criteria for accurate diagnosis may differentiate WDPMT from mesotheliomas.

Pathogenic germline alterations were found in 24% (n = 4) of patients with WDPMT; three had a personal history of other malignancies, including skin, GI, and ovarian cancers. Two patients had alterations in *RAD51X*. *RAD51C* alterations are associated with familial predisposition for ovarian and breast cancers, whereas *RAD51D* alterations are associated with breast, ovarian, prostate, and GI cancers.^12^ One patient had a pathogenic alteration in *TGFBR1*, which occurs in connective tissue/skeletal conditions.^13^ One patient had an *MSH-6* alteration, which is associated with Lynch syndrome. This is the first report of an association between these inherited alterations and WDPMT. Germline testing is recommended for patients with DPM and PM.^7,8^ Given that the germline alterations in WDPMT mostly occurred in patients with other concurrent malignancies, there is a potential diagnostic bias. In routine clinical care, germline testing should be considered for patients with WDPMT and concurrent malignancies. We propose prospective germline evaluation of all patients with WDPMT to better define alteration rates.

Our study has several limitations, the most significant of which is selection bias. Patients were identified at a tertiary academic cancer center, and most patients were diagnosed with WDPMT incidentally during management of other malignancies, which may bias the frequency of germline alterations. A larger prospective study is needed to determine the WDPMT populations’ prevalence of inherited alterations.

Our findings illustrate the importance of the expert delineation of WDPMT from more aggressive malignant processes, such as PM. Proper pathologic workup is of the utmost importance. NGS can help to differentiate WDPMT from lesions with malignant potential that may require more aggressive intervention. Given the indolent nature of WDPMT, no established role for cytoreductive surgery or systemic therapy, and excellent disease-specific outcomes, WDPMT can likely be monitored with expectant management under the guidance of a multidisciplinary oncology team without aggressive upfront interventions. This series, the largest of its kind, should offer reassurance to surgeons, oncologists, and patients that active surveillance is the optimal route though duration of observation remains unknown.

## Supplementary Information

Below is the link to the electronic supplementary material.

Supplementary file1 (DOCX 830 kb)
