# Supplementary material for: Clinical Characteristics and Outcomes of Patients with Well-Differentiated Papillary Peritoneal Mesothelial Tumors
Source: Ann Surg Oncol. 2024 Aug 21;31(12):7973–7. doi: 10.1245/s10434-024-16004-2 (PMC11466981; doi:10.1245/s10434-024-16004-2)
Supplement: Supplementary file 1 — Supplementary file1 (DOCX 830 kb) [file 10434_2024_16004_MOESM1_ESM.docx]

**Supplemental Table 1** Patient characteristics in the 19 patients with well-differentiated papillary peritoneal mesothelial tumors (WDPMT) that has comprehensive next-generation sequencing (NGS, MSK-IMPACT)

| **Patient characteristics** | **WDPMT (n = 19)** |
| --- | --- |
| **Median age at diagnosis (range)** | 54 (20-74) |
| **Sex**  **Male, n (%)**  **Female, n (%)** | 13 (68)  6 (32) |
| **Self-identified race**  **Caucasian, n (%)**  **African American, n (%)**  **Asian, n (%)**  **Other/Not disclosed, n (%)** | 14 (74)  3 (16)  2 (10)  0 |
| **Smoking status**  **Never, n**  **Ever, n (pack/yrs; range)** | 6  13 (8; 3-30) |
| **Incidentally diagnosed during other surgical procedure**  **No, n (%)**  **Yes, n (%)**  **Initial diagnostic surgery performed for:**  **GI cancers, n (%)**  **Colorectal cancer, n (%)**  **Esophageal cancer, n (%)**  **Gastric cancer, n (%)**  **Pancreatic cancer, n (%)**  **Metastatic breast cancers, n (%)**  **Ovarian neoplasms, n (%)**  **Prostate cancers, n (%)**  **Renal cell carcinomas, n (%)**  **Other malignancies, n (%)**  **Non cancer related surgeries, n (%)** | 1 (5)  18 (95)  5 (29)  3 (18)  0 (0)  1 (6)  1 (6)  0 (0)  0 (0)  6 (35)  0 (0)  2^a^ (12)  6^b^ (29) |
| **Disease-specific treatment**  **Surgical debulking for WDPMT, n (%)**  **Systemic therapies for WDPMT, n (%)** | 2 (4)  0 (0) |
| **Tumor mutational burden (mutations/Mb; median, range)** | 1 (0.8-1.6) |

*WDPMT* well-differentiated papillary peritoneal mesothelioma tumors; *mutations/Mb* mutations per megabase

^a^One liposarcoma and one gastrointestinal stromal tumor (GIST)

^b^One abdominal hysterectomy, one myomectomy, two sleeve gastrectomy, two diagnostic laparoscopies

**Supplemental Table 2** Patient characteristics of patients with well-differentiated papillary peritoneal mesothelial tumors (WDPMT; n = 54) and a previously published cohort of patients with peritoneal mesotheliomas^7^ (PM; n = 50)

| **Patient characteristics** | **WDPMT**  **(n = 54)**  **n (%)** | **PM**  **(n = 50)**  **n (%)** | ***p*** |
| --- | --- | --- | --- |
| **Median age at diagnosis (range)** | 55 (20 - 76) | 62 (23, 77) | 0.09^a^ |
| **Sex**  **Male**  **Female** | 27 (50)  27 (50) | 22 (44)  28 (56) | 0.07b |
| **Smoking status**  **Never, n**  **Ever, n (pack/yrs; range)** | 29  25 (8; 3-50) | 36  14 (10; 3-32) | 0.56^b^ |
| **Extra-abdominal metastases**  **Lung parenchyma/thoracic adenopathy**  **Pleura**  **Soft tissue** | 0  --  --  -- | 14 (28)  9 (18)  3 (6)  2 (4) | -- |
| **Treatment**  **Disease-specific surgical debulking**  **Disease-specific infusional therapies**  **IV cytotoxic chemotherapy**  **IP cytotoxic chemotherapy**  **Immunotherapy** | 2 (4)  0  --  --  -- | 37 (74)  44 (88)  39 (78)  35 (70)  15 (30) | -- |

*IV* intravenous; *IP* intraperitoneal

^a^Mann-Whitney *U* test

^b^Fisher’s exact test

**Supplemental Table 3** Comparison of genomic alterations of patients with well-differentiated papillary peritoneal mesothelial tumors (WDPMT; n=19), peritoneal mesotheliomas^7^ (PM; n = 50) with next-generation sequencing (NGS) on the MSK-IMPACT platform as well as diffuse pleural mesotheliomas^11^ (DPM, The Cancer Genome Atlas [TCGA]; n = 74) from a publicly available mesothelioma data set. All *p*-values were calculated by Fisher’s exact method. Bold denotes *p* ≤ 0.05

| **Alteration** | **WDPMT**  **(n = 19)**  **(%)** | **PM**  **(n = 50)**  **(%)** | **DPM (n = 74)**  **(%)** | **WDPMT vs. PM**  ***p* value** | **WDPMT vs. DPM**  ***p* value** | **DPM vs. TCGA**  ***p* value** |
| --- | --- | --- | --- | --- | --- | --- |
| *TRAF7* | 17 (89) | 0 | 0 | **<0.0001** | **<0.0001** | 1.00 |
| *CREBBP* | 2 (11) | 2 (4) | 1 (1.4) | 0.30 | 0.10 | 0.56 |
| *CDKN2A/B* | 0 | 4 (8) | 28 (33) | 0.57 | **0.0005** | **0.0001** |
| *BAP1* | 0 | 30 (60) | 34 (46) | **0.001** | **<0.0001** | 0.14 |
| *NF2* | 0 | 12 (24) | 23 (31) | **0.03** | **0.001** | 0.22 |
| *SETD2* | 0 | 11 (22) | 11 (15) | **0.03** | 0.11 | 0.34 |
| *TP53* | 0 | 8 (16) | 10 (14) | 0.10 | 0.20 | 0.80 |
| *LATS2* | 0 | 4 (8) | 9 (12) | 1.00 | 0.68 | 0.56 |
| *TERT* | 0 | 2 (4) | 3 (4) | 1.00 | 1.00 | 1.00 |


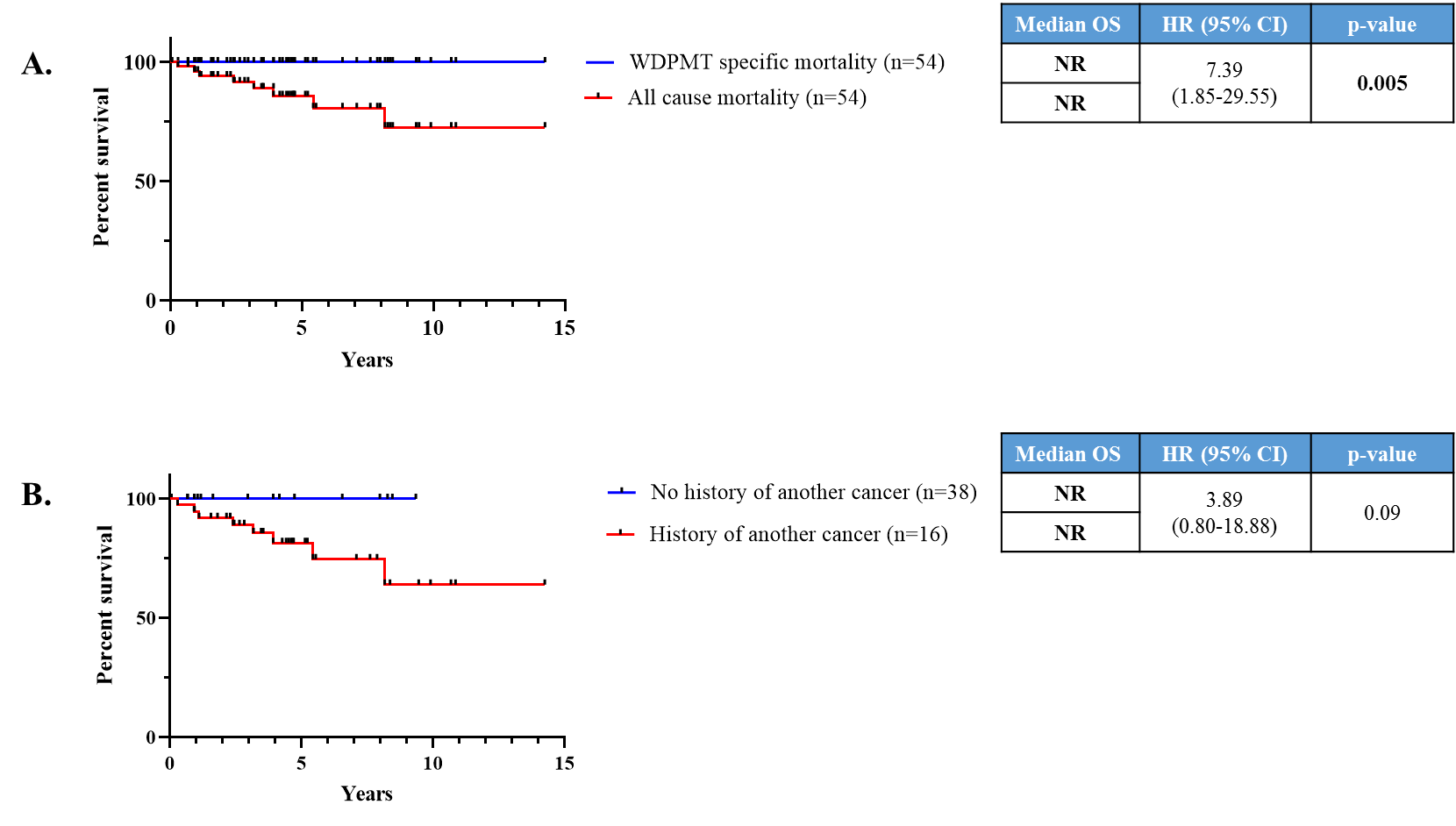


**Supplemental Fig. 1** **A** Overall survival (OS) of 54 patients with well-differentiated papillary peritoneal mesothelial tumors (WDPMT) based on all-cause mortality and WDPMT-specific mortality was not reached (NR, median follow up 4.5 years; range 0.1-14.2 years). **B** OS based on presence or absence of a personal history of another malignancy was not reached. *WDPMT* well-differentiated papillary peritoneal mesothelial tumors; *OS* overall survival; *HR* hazard ratio; *CI* confidence interval; *NR* not reached

**
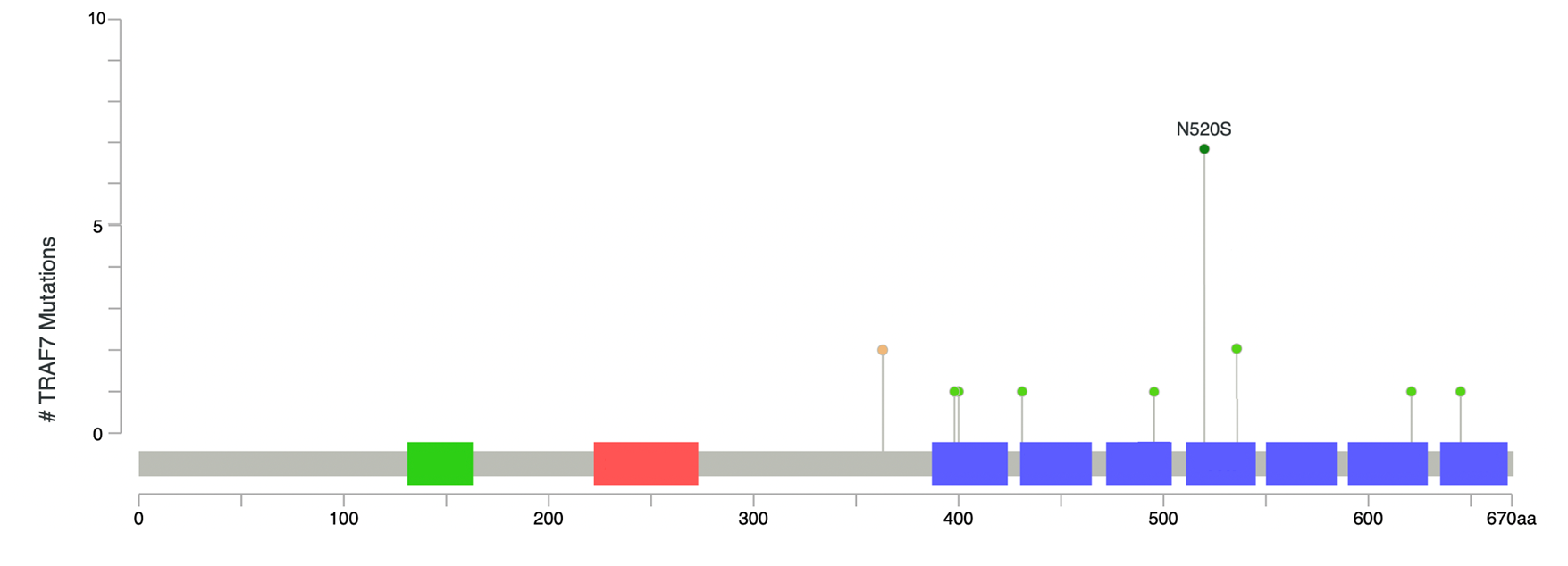
**

**Supplemental Fig. 2** Lollipop diagram depicting the distribution of the 17 *TRAF7* alterations noted in the 19 samples obtained from well-differentiated papillary peritoneal mesothelial tumors (WDPMT) noting enrichment in the WD-40 domain. Blue (WD-40), Red (zf-TRAF), Green (zf-C3HC4)


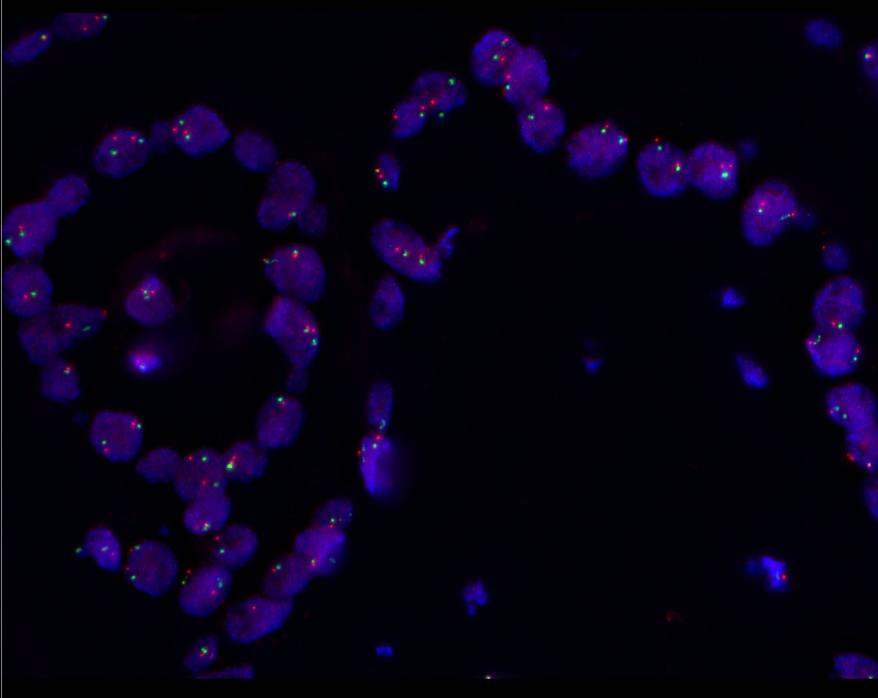


**Supplemental Fig. 3** Confirmatory analysis of a sample called on next-generation sequencing as having a potential *CDKN2A* deep deletion. CDKND1 (9p21) fluorescence in situ hybridization (FISH) probe (orange) used in combination with a centromere specific probe for chromosome 9 (green, CEP9) to examine CDKN2A copy status. Homozygous deletion of *CDKN2A* is indicated if cells show no signals for the *CDKN2A* and two or more signals for the internal control probe CEP9. Representative FISH images were captured using a Zeiss fluorescence microscope coupled with Metasystems ISIS software (Newton, MA). FISH showed two copies of CDKN2A confirming that the initially reported copy call was a false-positive call
